# Impact of *DIO2* polymorphisms on quality of life and TSH suppression therapy in patients with papillary thyroid cancer

**DOI:** 10.1186/s12902-025-02085-x

**Published:** 2025-12-02

**Authors:** Junhan Chen, Zhiqing Lin, Yezhe Luo, Hui Tang, Fangsen Chen, Peitian Liu, Yanling Huang

**Affiliations:** 1 Department of Endocrinology and Metabolism, School of Medicine, Zhongshan Hospital of Xiamen University, Xiamen University, Xiamen, China; 2 The School of Clinical Medicine, Fujian Medical University, Fuzhou, China; 3 Department of Pediatrics, School of Medicine, Zhongshan Hospital of Xiamen University, Xiamen University, Xiamen, China; 4 Department of General Surgery, School of Medicine, Zhongshan Hospital of Xiamen University, Xiamen University, Xiamen, China; 5 Department of Nuclear Medicine, School of Medicine, Zhongshan Hospital of Xiamen University, Xiamen University, Xiamen, China

**Keywords:** Papillary thyroid cancer, TSH-Suppressive therapy, Type 2 deiodinase, Single nucleotide polymorphisms, Quality of life assessment

## Abstract

**Background:**

This study aims to explore the impact of type 2 deiodinase (*DIO2*) polymorphisms on TSH suppression therapy and quality of life in patients with papillary thyroid cancer.

**Methods:**

A cohort of 200 patients with papillary thyroid cancer was recruited at Zhongshan Hospital, Xiamen University, between September 2021 and September 2022, with 88 in the lobectomy group and 74 in the total thyroidectomy plus ^131^I treatment group. Clinical data were collected, and quality of life was evaluated using the EQ-5D-3L scale. Genetic polymorphisms rs225014 and rs12885300 of *DIO2* were analyzed from whole blood samples.

**Results:**

In comparison between the two surgical treatments, the total thyroidectomy plus ^131^I treatment group had lower scores in usual activities (*P* = 0.016), anxiety/ depression (*P* < 0.001), EQ-5D index (*P* < 0.001), and EQ VAS (*P* < 0.001) than the lobectomy group. In the lobectomy group, *DIO2* rs225014 and rs12885300 polymorphisms did not significantly impact quality of life, thyroid hormone levels, or replacement therapy doses. However, in the total thyroidectomy plus ^131^I treatment group, patients with *DIO2* rs225014 wild type (AA) exhibited higher EQ-5D index scores than those with mutant types (AG, GG) (*P* = 0.025). In a multiple linear regression model, using L-T4 Dose/Weight as the dependent variable, *DIO2* rs225014 mutant types (AG, GG) were positively correlated with L-T4 Dose/Weight (β = 0.244, *P* = 0.028).

**Conclusion:**

Patients in the lobectomy group exhibited higher quality of life than those in the total thyroidectomy plus ^131^I treatment group. In the latter, *DIO2* rs225014 mutant genotypes (AG, GG) showed a modestly lower EQ-5D index score and a slight association with higher L-T4 dose/weight ratios. No significant differences were found for *DIO2* rs12885300. These findings, limited by sample size and effect size, require validation in larger, multicenter studies.

**Supplementary Information:**

The online version contains supplementary material available at 10.1186/s12902-025-02085-x.

## Introduction

Papillary thyroid cancer (PTC) is the most prevalent type of thyroid cancer, representing approximately 90% of all cases. In recent years, the incidence of PTC has increased significantly [1, 2]. Surgery is the primary initial treatment for PTC, as it reduces the risk of recurrence and metastasis by excising the tumor, surrounding tissues, and lymph nodes through a standardized procedure. This intervention also establishes the foundation for clinical staging and stratification of recurrence risk. Postoperative evaluations and subsequent therapies are essential for minimizing recurrence and enhancing prognosis. Based on the recurrence risk stratification, the majority of thyroid cancer patients require TSH suppression therapy post-surgery [3]. The extent of TSH suppression is closely associated with the prognosis of PTC, and for many patients, this therapy may be a lifelong necessity. While most patients can restore normal thyroid hormone levels and maintain TSH level within the target range through appropriate L-T4 replacement therapy [4], some experience elevated FT4 levels but still fail to achieve the target TSH level [5]. These patients not only confront challenges related to the tumor but also experience adverse cardiac and skeletal effects associated with supraphysiological doses of T4 [6].

Levothyroxine (T4, 3,5,3’,5’-triiodo-L-thyronine, L-T4) is the preferred agent for TSH suppression therapy, as endorsed by national and international guidelines. Following oral administration, L-T4 is converted to T3 by deiodinases, restoring adequate T3 and T4 levels in thyroid cancer patients and suppressing serum TSH levels [5]. Oral L-T4 therapy is influenced by various factors, including age, diet, comorbidities, treatment adherence, digestive disorders, and deiodinase activity [7]. Deiodinases are a group of oxidoreductases involved in the synthesis, release, and inactivation of thyroid hormones. There are three isoforms of deiodinase: type 1 (D1), type 2 (D2), and type 3 (D3) [8]. These deiodinases act on the iodine atoms in the phenol ring (activation) or tyrosine ring (deactivation) of T4 and T3, regulating thyroid hormone levels at the cellular level [9]. D3, located in the cell membrane, inactivates T3 and T4 through the deiodination of the tyrosine ring. D1 primarily contributes to serum T3 production, while D2 regulates the local conversion of T4 to T3 in tissues, particularly in the hypothalamus and pituitary, playing a crucial role in the negative feedback regulation of TSH secretion [10].

Single nucleotide polymorphisms (SNPs) in the *DIO2* gene can modify D2 activity, impacting the efficiency of T4 to T3 conversion and potentially affecting the effectiveness of TSH suppression therapy in post-surgical PTC patients. Several studies have investigated the impact of *DIO2* gene polymorphisms on L-T4 replacement therapy in hypothyroid patients [11–13]. The role of *DIO2* polymorphisms, particularly at loci rs225014 (Thr92Ala) and rs1285300 (ORFa-Gly3Asp), has been the subject of several studies investigating their effects on thyroid hormone regulation and treatment outcomes in hypothyroid patients. *DIO2* rs225014 may influence D2 activity, resulting in lower T3 concentrations in the blood [14]. *DIO2* rs12885300, can affect the set point of the hypothalamic-pituitary-thyroid axis, altering the negative feedback of T4 on TSH [15]. But some studies have not found a significant correlation between the efficacy of TSH suppression therapy and *DIO2* gene polymorphisms [16]. The impact of *DIO2* gene polymorphisms on hypothyroidism treatment, particularly regarding TSH suppression therapy following papillary thyroid cancer surgery, remains unclear and necessitates further investigation.

## Methods

### Patients and study design

This study was a single-center prospective cohort study conducted at Zhongshan Hospital, Xiamen University in Fujian Province, China. The study received approval from the Ethics Committee of Zhongshan Hospital, Xiamen University (Ethics Approval Number: xmzsyy 2021 − 123). This study was registered in the National Medical Research Registration and Filing Information System of China (https://www.medicalresearch.org.cn) in May 2021 (Registration No: MR-35-21-015058) and in ClinicalTrials.gov in February 2022 (Registration No: NCT05247476). Patients diagnosed with papillary thyroid cancer (PTC) and admitted to Zhongshan Hospital, Xiamen University, between September 2021 and September 2022 were included in the study. Participants were divided into two groups: 100 patients who underwent unilateral lobectomy and isthmectomy (lobectomy group), and 100 patients who underwent total thyroidectomy with central lymph node dissection followed by ^131^I therapy (total thyroidectomy plus ^131^I treatment group).

Inclusion Criteria: (1) Age between 18 and 70 years with complete preoperative medical records; (2) Underwent either unilateral lobectomy with isthmectomy or total thyroidectomy with central lymph node dissection combined with ^131^I therapy; (3) Postoperative pathology confirmed papillary thyroid cancer.

Exclusion Criteria: (1) Incomplete preoperative medical records; (2) Postoperative pathology confirming other malignant tumors or different types of thyroid cancer; (3) Previous thyroid surgery; (4) History of head and neck radiotherapy; (5) Use of medications that may interfere with thyroid hormone treatment; (6) Poor adherence to T4 therapy; (7) Pregnant women, psychiatric patients, renal impairment (GFR < 60 ml/min), or chronic liver disease.

At enrollment, patients were assessed for body weight, lipid profile, renal function, electrolyte levels, TSH, FT3, FT4, TPOAb, TG, and other relevant markers. Enrolled patients received levothyroxine for 6 months and attended regular outpatient follow-ups to monitor their thyroid hormone levels. The T4 dose was adjusted according to ATA guidelines to maintain TSH levels within the target range (0.5-2.0 mU/L for low-risk patients in the lobectomy group and < 0.1 mU/L for the total thyroidectomy plus ^131^I treatment group). TSH suppression therapy was administered for a duration of 6 months. The EQ-5D-3L quality of life questionnaire was used to assess the health status of patients during follow-up. Throughout the follow-up period, levothyroxine tablets (50 µg, Merck KGaA, Darmstadt) were prescribed by an endocrinologist. Ultimately, 29 patients were excluded due to loss to follow-up, and 9 were excluded due to medication irregularities, resulting in a total of 162 patients (88 in the lobectomy group and 74 in the total thyroidectomy plus ^131^I treatment group).

### Genotyping

A sample of peripheral blood (5 ml) was collected from the patients in EDTA-containing tubes for genomic DNA extraction using the CWE9600 Blood DNA Kit. The genotyping assay was conducted by KingMed Diagnostics Co. using kompetitive allele specific PCR (KASP). The details of the KASP primers are summarized in Table 1.The fluorescent group sequence of primer FAM was: 5’ GAAGGTCGGAGTCAAC.

**Table 1:** The KASP primers of rs225014 and rs12885300

Gene loci	Primer name	Primer sequence
rs225014	Primer Allele FAM	GAAGGTCGGAGTCAACGGATTCAGTGTGGTGCATGTCTCCAGTA
	Primer Allele HEX	GAAGGTGACCAAGTTCATGCTAGTGTGGTGCATGTCTCCAGTG
	Reverse primer	GCATGTGGCTCCCTCAGCTAT
rs12885300	Primer Allele FAM	GAAGGTCGGAGTCAACGGATTGTTTAAAGAGCATAGAGACAATGAAAGG
	Primer Allele HEX	GAAGGTGACCAAGTTCATGCTCGTTTAAAGAGCATAGAGACAATGAAAGA
	Reverse primer	CCGTTAAAGACAGGCAGTTCTACTTTC

GGATT3’, while the fluorescent group sequence for primer HEX was 5’ GAAGGTGACCAAGTTCATGCT 3’. The primers were synthesized by Sangon Biotech (Shanghai) Co., Ltd. A primer mix containing KASP primers and 2 × KASP Master -Mix was prepared, mixed with the dried DNA, and subsequently amplified by PCR in a high-throughput water bath system (Hydrocycler). Fluorescence signals were detected by the Pherastar SNP typing detector to assess the genotyping status of the gene loci. SNP typing detector to assess the genotyping status of the gene loci. 5%of the total samples were genotyped again, with a concordance rate of 100% among the repeated cases, indicating that the genotyping results were reliable.

### Clinical and laboratory evaluations

We collected demographic and clinical characteristics, including age, sex, and weight. Routine biochemical indices, including albumin, globulin, triglycerides, total cholesterol, high-density lipoprotein, low-density lipoprotein, apolipoprotein-A, apolipoprotein-B, glucose, creatinine, and potassium, were measured using the Siemens ADVIA2400 automatic biochemical analyzer. The levels of free triiodothyronine (FT3), free thyroxine (FT4), and thyroid-stimulating hormone (TSH) were measured using the i2000SR fully automated chemiluminescence immunoassay system.

### EQ-5D-3L

This study uses the EQ-5D-3L questionnaire to assess the quality of life in patients with thyroid cancer (Table S1). The EQ-5D-3L consists of two main components: the EQ-5D descriptive system and the EQ-5D visual analogue scale (EQ VAS). The descriptive system includeed five dimensions: mobility, self-care, usual activities, pain/discomfort, and anxiety/depression. Each dimension was categorized into three levels: no problems, some problems, and extreme problems (labeled 1–3). The visual analogue scale (VAS) records the respondent’s self-rated health status on a vertical scale, with endpoints marked as “best health status” and “worst health status.” The EQ VAS provided a quantitative measure of health outcomes, ranging from 0 (worst health) to 100 (best health). Data from the five health dimensions in the EQ-5D-3L can be converted into a utility index, known as the EQ-5D index (Table S2), representing overall health status. The collected data were subjected to comprehensive statistical analysis from three perspectives: health dimensions, EQ VAS, and EQ-5D index, to assess the quality of life in thyroid cancer patients.

### Statistical analysis

Given the limited sample size for genetic studies, heterozygotes (AG) and homozygotes (GG for rs225014; AA for rs12885300) were combined for statistical analysis to enhance power and ensure robust comparisons. It is a common strategy in genetic association studies to increase statistical power, particularly when minor allele frequencies are low. This approach mitigates underpowered analyses for rare homozygous genotypes but may obscure genotype-specific effects between heterozygotes and homozygotes. Continuous variables were expressed as mean ± SD and analyzed using ANOVA or independent samples t-test. Categorical variables were expressed as percentages and analyzed using the chi-square test. Pearson correlation was used for univariate analysis, and statistically significant factors were further examined using multiple linear regression. Differences were considered statistically significant at *P* < 0.05.

## Results

### Baseline characteristics

Table 2 summarized the demographic characteristics of the 162 subjects enrolled in this study and examines statistical differences between subgroups at baseline. In both the lobectomy and total thyroidectomy plus ^131^I groups, no significant differences were observed in age, sex, blood lipids, renal function, or liver function between the wild-type and mutant genotypes of *DIO2* rs225014 and *DIO2* rs12885300, suggesting the two groups were comparable.

**Table 2:** Baseline characteristics of PTC patients

	The lobectomy group						The total thyroidectomy with ^131^I treatment group					
	rs225014			rs12885300			rs225014			rs12885300		
genotype	AA	AG、GG	*P*	GG	AA、AG	*P*	AA	AG、GG	*P*	GG	AA、AG	*P*
Participants(n)	30	58		59	29		25	49		51	23	
Age (years)	40.7 ± 11.15	43.55 ± 11.61	0.271	42.81 ± 11.94	42.1 ± 10.63	0.787	39 ± 12.67	40.2 ± 12.39	0.696	39.14 ± 11.72	41.26 ± 13.98	0.499
Gender	70.00%	67.24%	0.795	72.89%	58.62%	0.181	52.00%	59.18%	0.558	62.75%	43.48%	0.124
Weight (kg)	63.55 ± 14.19	63.88 ± 9.63	0.898	64.02 ± 11.96	63.26 ± 10.04	0.769	68.48 ± 14.16	62.23 ± 12.58	0.057	62.32 ± 13.08	68.84 ± 13.19	0.051
TSH (mIU/L)	3.02 ± 1.41	2.57 ± 1.31	0.106	2.64 ± 1.24	2.89 ± 1.27	0.380	3.18 ± 1.13	2.84 ± 1.15	0.233	2.94 ± 1.13	2.99 ± 1.21	0.864
FT3 (pmol/L)	5.34 ± 1.47	5.52 ± 1.27	0.540	5.39 ± 1.43	5.60 ± 1.12	0.501	5.12 ± 1.35	5.05 ± 0.99	0.802	5.03 ± 1.08	5.17 ± 1.20	0.628
FT4 (pmol/L)	16.70 ± 2.62	15.79 ± 3.24	0.189	16.04 ± 3.27	16.21 ± 2.63	0.810	16.76 ± 2.82	17.16 ± 2.81	0.533	16.81 ± 2.68	17.51 ± 3.07	0.326
Albumin (g/L)	43.93 ± 6.89	44.56 ± 6.14	0.666	44.99 ± 7.24	43.02 ± 3.87	0.173	41.08 ± 2.63	42.1 ± 2.77	0.132	41.55 ± 2.62	42.21 ± 3.02	0.339
Globulin (g/L)	30.49 ± 4.77	30.33 ± 4.42	0.876	30.28 ± 5.03	30.61 ± 3.32	0.751	27.88 ± 3.12	29.35 ± 3.38	0.074	29.23 ± 3.28	28.01 ± 3.41	0.148
TG (mmol/L)	1.19 ± 0.52	1.4 ± 1.13	0.341	1.44 ± 1.14	1.1 ± 0.38	0.116	1.78 ± 0.98	2.28 ± 2.88	0.401	2.24 ± 2.82	1.84 ± 1.05	0.51
TC (mmol/L)	4.61 ± 0.66	4.92 ± 0.99	0.126	4.83 ± 0.92	4.78 ± 0.86	0.785	5.82 ± 1.12	6.1 ± 1.26	0.351	6.17 ± 1.27	5.65 ± 1.03	0.087
HDL (mmol/L)	1.18 ± 0.27	1.21 ± 0.26	0.541	1.21 ± 0.28	1.19 ± 0.23	0.767	1.44 ± 0.29	1.54 ± 0.39	0.276	1.54 ± 0.39	1.43 ± 0.27	0.231
LDL (mmol/L)	3.15 ± 0.54	3.38 ± 0.81	0.170	3.3 ± 0.76	3.3 ± 0.7	0.995	4 ± 0.92	4.04 ± 0.86	0.858	4.11 ± 0.89	3.84 ± 0.83	0.22
Apolipoprotein A (g/L)	1.29 ± 0.18	1.37 ± 0.19	0.073	1.35 ± 0.19	1.31 ± 0.2	0.347	1.49 ± 0.21	1.67 ± 0.64	0.197	1.66 ± 0.63	1.48 ± 0.22	0.177
Apolipoprotein B (g/L)	0.88 ± 0.15	0.93 ± 0.23	0.271	0.91 ± 0.21	0.92 ± 0.21	0.918	1.12 ± 0.29	1.12 ± 0.29	0.959	1.15 ± 0.29	1.06 ± 0.28	0.196
Glucose (mmol/L)	5.47 ± 0.58	5.66 ± 1.48	0.495	5.5 ± 0.68	5.79 ± 1.96	0.318	4.85 ± 0.72	4.94 ± 0.57	0.593	4.9 ± 0.58	4.93 ± 0.71	0.839
Creatinine (umol/L)	63.19 ± 16.17	63.58 ± 16.77	0.915	62.02 ± 15.29	66.36 ± 18.61	0.248	77.47 ± 19.95	71.83 ± 17.28	0.212	72.04 ± 18.53	77.48 ± 17.56	0.239
Potassium (mmol/L)	4.11 ± 0.25	4.09 ± 0.32	0.731	4.04 ± 0.24	4.21 ± 0.37	0.013	3.96 ± 0.3	3.99 ± 0.32	0.627	3.99 ± 0.31	3.97 ± 0.33	0.847

Note: Abbreviations: TG: triglycerides, TC: total cholesterol, HDL: high-density lipoprotein-cholesterol, LDL: low-density lipoprotein-cholesterol

### Hardy-Weinberg equilibrium

The genotype and allele frequencies of the two *DIO2* polymorphisms were tested for Hardy-Weinberg equilibrium, confirming that the study population was in genetic equilibrium. The genotype distributions of *DIO2* rs225014 (*P* = 0.839) and *DIO2* rs12885300 (*P* = 0.870) did not significantly deviate from the expected frequencies (Table 3).

**Table 3:** Hardy-Weinberg equilibrium test

	Gene	Frequency	Genotype	Patient Genotype Frequency	Theoretical Genotype Frequency	*P*
rs225014	A	60.12%	AA	34.36%	36.14%	0.839
	G	39.88%	AG	51.53%	47.95%	
			GG	14.11%	15.90%	
rs12885300	G	83.13%	GG	68.10%	69.11%	0.870
	A	16.87%	AG	30.06%	28.05%	
			AA	1.84%	2.85%	

### Impact of different treatment on quality of life

**Fig. 1:**
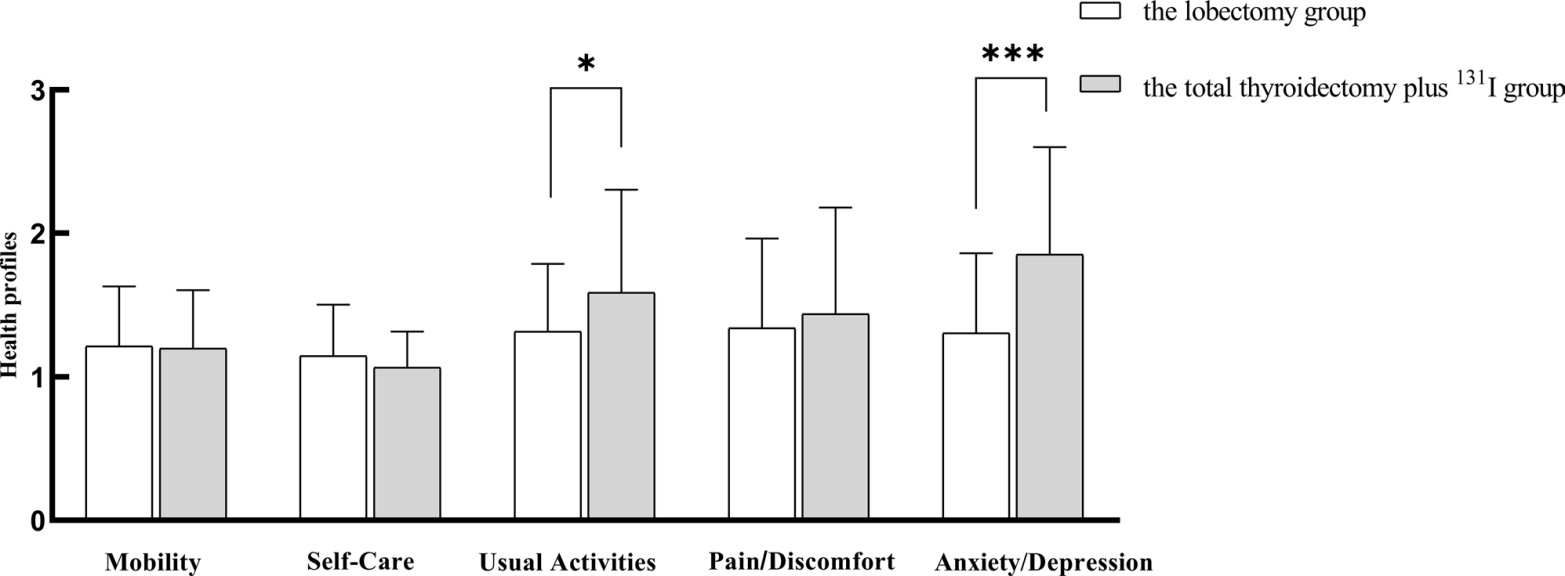
Impact of different treatment on quality of life. Note: Fig. 1 shows that the lobectomy group demonstrated significantly better quality of life scores compared to the total thyroidectomy plus ^131^I treatment group in the dimensions of usual activities (* indicates *P* < 0.05) and anxiety (*** indicates *P* < 0.001)

As illustrated in Fig. 1, there were no significant differences between the two treatment groups in the dimensions of mobility, self-care, and pain. However, in the dimensions of usual activities (*P* = 0.016) and anxiety (*P* < 0.001), the lobectomy group demonstrated significantly better quality of life scores compared to the total thyroidectomy plus ^131^I treatment group. The differences in EQ VAS and EQ-5D index between the two groups are summarized in Table 4. The lobectomy group had significantly higher scores on quality of life metrics.

**Table 4:** The difference of EQ-VAS and EQ-5D index between the lobectomy group and the thyroidectomy with ^131^I treatment group

	The lobectomy group	The total thyroidectomy with ^131^I treatment group	z	*P*
EQ-5D index	0.832(0.739,0.859)	0.745(0.626,0.823)	-4.268	<0.001
EQ VAS	85(80,90)	70(60,80)	-4.894	<0.001

Note: Abbreviations: EQ-5D: EuroQol Five Dimensions; EQ-5D index༚Data from the five health dimensions in the EQ-5D-3L can be converted into a utility index, known as the EQ-5D index, representing overall health status. the unified utility index for assessing general health; EQ VAS: EQ-5D visual analogue scale

### Impact of *DIO2* gene polymorphism on quality of life

**Fig. 2:**
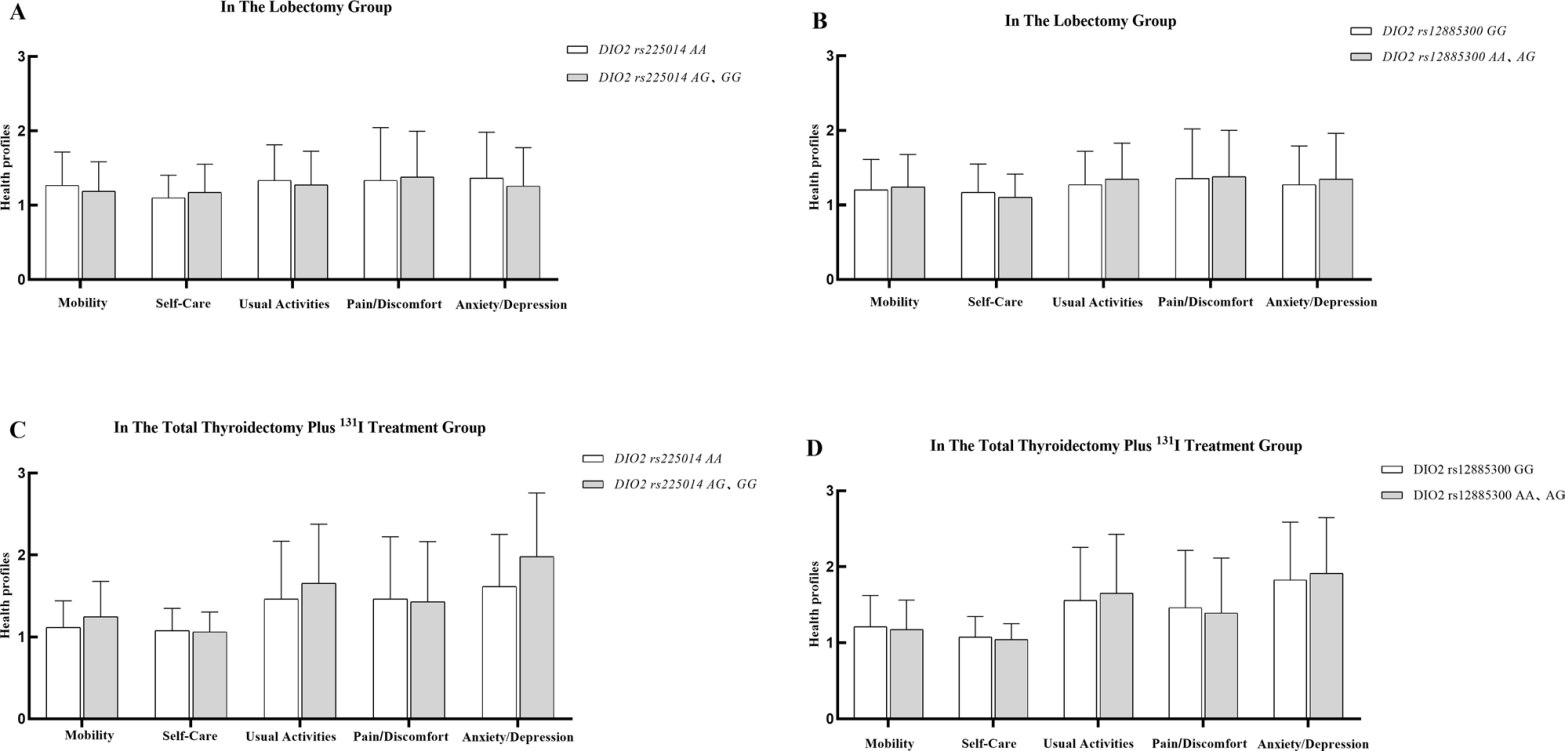
Impact of *DIO2* gene polymorphism on quality of life. Note: In the lobectomy group, the differences in EQ-5D-3L scores for various genotypes of *DIO2* rs225014 are presented in Fig. 2A, and the differences for *DIO2* rs12885300 genotypes are shown in Fig. 2B. In the total thyroidectomy plus ^131^I treatment group, the differences in EQ-5D-3L scores among various genotypes of *DIO2* rs225014 are presented in Fig. 2C, while those for *DIO2* rs12885300 are shown in Fig. 2D

To ensure sufficient statistical power given the limited sample size, heterozygotes and homozygotes for *DIO2* rs225014 (AG + GG) and *DIO2* rs12885300 (AA + AG) were combined for comparison against their respective wild-type genotypes (AA for rs225014; GG for rs12885300). This approach was necessary to achieve reliable statistical comparisons but may obscure potential differences in the effects of heterozygous versus homozygous genotypes. Figure 2 illustrates the differences in EQ-5D-3L scores among different genotypes of *DIO2* rs225014 and *DIO2* rs12885300 in the lobectomy and total thyroidectomy plus ^131^I treatment groups. In the lobectomy group, no differences were observed in EQ-5D-3L scores, EQ VAS, or EQ-5D index between the DIO2 rs225014 wild-type (AA) and mutant types (AG, GG) (Fig. 2A). Similarly, no differences were found in EQ-5D-3L scores, EQ VAS, or EQ-5D index between the *DIO2* rs12885300 wild-type (GG) and mutant types (AA, AG) (Fig. 2B). In the total thyroidectomy plus ^131^I treatment group, the *DIO2* rs225014 wild-type (AA) had a higher EQ-5D index score (*P* = 0.04) than the mutant types (AG, GG) (Table 5), but no differences were observed in EQ-5D-3L scores or EQ VAS between the wild-type (AA) and mutant types (AG, GG) (Fig. 2C). Similarly, for *DIO2* rs12885300, no differences were found in EQ-5D-3L scores, EQ VAS, or EQ-5D index between the wild-type (GG) and mutant types (AA, AG) (Fig. 2D).

**Table 5:** The difference in genotypic subtypes of *DIO2* rs225014 and rs12885300 in terms of EQ-VAS and EQ-5D index

	225,014			12,885,300		
Genotype	AA	AG、GG	*P*	GG	AA、AG	*P*
**the lobectomy group**						
EQ VAS	85.5(70,90)	85(80,100)	0.452	80(80,100)	85(77.5,90)	0.860
EQ-5D index	0.83(0.69,0.89)	0.83(0.74,0.89)	0.940	0.85(0.74,1)	0.83(0.73,0.85)	0.275
**the total thyroidectomy with** ^**131**^**I treatment group**						
EQ VAS	80(60,90)	70(60,80)	0.264	80(60,80)	70(60,80)	0.581
EQ-5D index	0.77(0.63,0.85)	0.7(0.62,0.77)	0.025	0.76(0.63,0.82)	0.75(0.63,0.77)	0.686

Note: Abbreviations: EQ-5D index the unified utility index for assessing general health, EQ VAS EQ-5D visual analogue scale. Date shown as median and interquartile range

### The impact of *DIO2* gene polymorphisms on thyroid hormone levels and L-T4 dosage

After six months of TSH suppression therapy, no significant differences were found between the wild-type and mutant types of *DIO2* rs225014 and *DIO2* rs12885300 in TSH levels, FT3 levels, FT4 levels, dosage, or weight-adjusted L-T4 dose (Dose/Weight) in either the lobectomy or total thyroidectomy plus ^131^I treatment groups (Table 6).

**Table 6:** The impact on TSH suppression therapy of different genotypes of *DIO2* rs225014 and rs12885300

	The lobectomy group				The total thyroidectomy with ^131^I treatment group			
	rs225014		rs12885300		rs225014		rs12885300	
Genotype	AA	AG、GG	GG	AA、AG	AA	AG、GG	GG	AA、AG
**TSH**,** mIU/L**	1.01(0.59,1.7)	1.28(0.74,1.92)	1.05(0.63,1.65)	1.36(0.95,2.09)	0.05(0.01,0.27)	0.05(0.02,0.3)	0.05(0.02,0.26)	0.12(0.02,0.50)
**Z**		-1.017		-1.753		-0.606		-1.168
***P***		0.309		0.08		0.545		0.243
**FT3**,**pmol/L**	4.8(4.25,5.46)	4.54(4.09,4.86)	4.58(4.12,5.09)	4.66(4.23,5.03)	5.53(4.52,6.41)	5.34(4.69,6.51)	5.49(4.71,6.50)	5.44(4.54,6.52)
**Z**		-1.787		-0.241		-0.189		-0.345
***P***		0.074		0.809		0.850		0.730
**FT4**,** pmol/L**	20.31(18.12,21.46)	18.22(16.97,20.66)	18.75(17.43,21.29)	18.85(16.64,20.67)	26.17(22.31,29.66)	26.44(22.63,28.62)	26.44(22.69,29.51)	26.17(21.96,28.21)
**Z**		-2.007		-0.768		-0.183		-0.625
***P***		0.052		0.397		0.855		0.532
**Dose**,** ug**	73.75(50,87.5)	57.72(50,78.13)	62.5(50,87.5)	62.5(50,83.75)	112.50(100,131.25)	114.28(100,134.51)	112.5(100,133.33)	120(100,137.5)
**Z**		-0.842		-0.111		-0.199		-0.537
***P***		0.400		0.912		0.843		0.591
**Dose/Weight**, **ug/kg**	1.15(0.89,1.43)	0.95(0.83,1.38)	1.02(0.85,1.37)	1(0.81,1.4)	1.79(1.52,1.93)	1.83(1.70,2.08)	1.82(1.67,2.04)	1.81(1.55,1.96)
**Z**		-1.211		-0.297		-1.887		-1.396
***P***		0.226		0.766		0.059		0.163

Note: Abbreviations: TSH: thyroid stimulating hormone, FT3: free triiodothyronine, FT4: free thyroxine, Dose/Weight: the ratio of levothyroxine dose to body weight

### Univariate regression and multiple linear regression analysis

To further evaluate the factors influencing TSH suppressive therapy, we used L-T4 Dose/Weight as the dependent variable. In the lobectomy group, initial univariate analyses revealed no association between L-T4 Dose/Weight and age (*P* = 0.988), gender (*P* = 0.258), *DIO2* rs12885300 SNPs (*P* = 0.946), *DIO2* rs225014 SNPs (*P* = 0.415), TSH (*P* = 0.274), FT3 (*P* = 0.158), or FT4 (*P* = 0.478) (Table 7). In the total thyroidectomy plus ^131^I treatment group, initial univariate analyses showed that age (*P* = 0.020), *DIO2* rs225014 SNPs (*P* = 0.027), and *DIO2* rs12885300 SNPs (*P* = 0.045) were associated with L-T4 Dose/Weight (Table 7). Since TSH (*P* = 0.947), FT3 (*P* = 0.338), and FT4 (*P* = 0.056) levels are clinically related to L-T4 Dose/Weight, they were included in the multivariate regression model. The covariates associated with L-T4 Dose/Weight in the final multivariate regression model, along with their respective regression coefficients and contributions, were listed in Table 8. The variance inflation factor for all six variables was below 10, indicating no multicollinearity. L-T4 Dose/Weight decreased with age and increased in patients with the mutant genotype (AG, GG) of *DIO2* rs225014 (β = 0.244, *P* = 0.028).

**Table 7:** Univariate analysis of L-T4 Dose/Weight ratio

variable	the lobectomy group			the total thyroidectomy with ^131^I treatment group	
	β	*P*	β		*P*
Age	-0.015	0.988	-2.383		0.020
Gender	1.138	0.258	0.366		0.715
rs225014	-0.819	0.415	2.253		0.027
rs12885300	-0.68	0.946	-2.039		0.045
TSH	1.101	0.274	-0.067		0.947
FT3	1.424	0.158	-0.965		0.338
FT4	-0.713	0.478	1.943		0.056

Note: Abbreviations: TSH: thyroid stimulating hormone, FT3: free triiodothyronine, FT4: free thyroxine

**Table 8:** Multivariate linear regression analysis of L-T4 Dose/Weight ratio

variates	B	SE	β	t	VIF	*P* value
Age	-0.008	0.004	-0.219	-1.963	1.124	0.054
rs225014	0.238	0.106	0.244	2.244	1.063	0.028
rs12885300	-0.159	0.114	-0.160	-1.400	1.173	0.166
TSH	0.073	0.140	0.069	0.519	1.582	0.606
FT3	-0.095	0.051	-0.232	-1.872	1.385	0.066
FT4	0.027	0.011	0.316	2.510	1.428	0.014
R2						25.8%
F						0.002

Note: Abbreviations: TSH: thyroid stimulating hormone, FT3: free triiodothyronine, FT4: free thyroxine

## Discussion

Thyroid cancer is the most common malignancy of the endocrine system. In recent years, the incidence of papillary thyroid cancer (PTC) has risen, drawing significant attention to treatment options and follow-up care. Most papillary thyroid carcinomas are low in malignancy, with a good postoperative prognosis and long-term survival. The effects of different surgical approaches and the extent of surgery on the quality of life in PTC patients have been well-documented. Lan Y’s study demonstrated that partial lobectomy led to better quality of life compared to total resection, with higher scores in physical and mental health assessments [17]. Similarly, Brooke Nickel’s study found that patients who underwent total thyroidectomy experienced more disturbances in daily life than those with partial lobectomy [18]. Mohamed A. also found that overall quality of life after total thyroidectomy was significantly worse than after adenoidectomy during the first postoperative year, although improvements were noted after one year [19]. Our study also found no significant differences between the adenoidectomy and total resection plus ^131^I treatment groups in mobility, self-care, and pain/discomfort, but in terms of usual activities, anxiety/depression the different is significant. Although exogenous thyroid hormone replacement can restore normal thyroid hormone levels in most patients, many continue to suffer from physical and mental discomfort, affecting both their work and mental well-being. Compared with total lobectomy plus ^131^I treatment, patients who underwent lobectomy had significantly better quality of life, as they retained part of the thyroid gland, which continues to produce T4 and T3. Additionally, patients who need to have total resection plus ^131^I treatment were classified as high risk for recurrence, and have to suppress TSH to < 0.1 mU/L. Some of these patients have higher-than-normal FT4 levels (mean 25.70 ± 5.32pmol/L).Supraphysiological doses of L-T4 make patients in a prolonged subclinical hyperthyroid state, further impacting their overall health. Therefore, adequate preoperative evaluation, appropriate surgical approach selection, and avoidance of overtreatment are critical to improving postoperative quality of life in PTC patients.

In the majority of postoperative thyroid cancer patients, lifelong TSH suppression therapy is required. L-T4 is converted into the more physiologically active T3 in peripheral tissues by deiodinase. Therefore, most patients require only L-T4 supplementation to reach physiological T4 and T3 levels and suppress TSH [20]. However, a small part of PTC patients fail to achieve the target TSH suppression, even with elevated T4 levels following adequate L-T4 treatment [21]. Approximately 5%-10% of patients continue to exhibit symptoms of hypothyroidism, such as fatigue, memory loss, and cold intolerance [7]. In patients with papillary thyroid cancer who are hypothyroid following total thyroidectomy, T3 production predominantly relies on the conversion of T4 in peripheral tissues. This process is largely regulated by type 2 deiodinase. The efficiency of this enzymatic activity becomes essential for managing thyroid hormone homeostasis in these patients. Type 2 deiodinase is also expressed in the hypothalamus and pituitary gland, where the locally converted T3 plays a key role in inhibiting TSH [22]. Thus, the efficacy of postoperative TSH suppression therapy in patients with PTC are closely linked to D2 activity. The *DIO2* gene, encoding type 2 deiodinase, is located on chromosome 14q24.3. Several single nucleotide polymorphisms (SNPs) for *DIO2* gene, including rs225014, rs12885300, rs225011, rs7140952, rs225012, and rs2839858, had been studied for their correlation with thyroid hormone levels, though most have shown no significant association. The most studied genetic polymorphisms are at the *DIO2* rs225014 and *DIO2* rs12885300 loci [13]. Zheng et al. found that patients with the *DIO2* rs225014 mutation had more difficulty achieving standard serum TSH levels, resulting in suboptimal TSH suppression therapy [23]. Research by Ramadhan et al. identified that abnormal D2 function plays a crucial role in achieving optimal TSH suppression in post-surgical PTC patients. This anomaly was also a primary cause of persistent hypothyroid symptoms, such as fatigue and lethargy, despite normal thyroid hormone levels [24]. Hoftijzer’s study suggested that the *DIO2* rs12885300 polymorphism may alter the set point of the hypothalamic-pituitary-thyroid axis, weakening the negative feedback regulation of TSH [15]. However, Santoro’s research showed no correlation between *DIO2* polymorphisms and TSH suppression therapy efficacy [16]. The relationship between DIO2 polymorphisms and the efficacy of TSH suppression therapy remains controversial, not only due to differences in sample sizes but also because of variations in study populations, genetic backgrounds, and environmental influences, etc. More robust multicenter studies are needed to clarify these genetic influences. In our study, we found that in the lobectomy group, residual thyroid tissue continued to synthesize and secrete T3, resulting in minimal dependence on D2 activity. Consequently, no variability was observed between the *DIO2* rs225014 and rs12885300 genotypes in terms of quality of life, thyroid hormone levels, or the L-T4 dose/weight ratio. In the group undergoing total thyroidectomy and ^131^I treatment, patients with the DIO2 rs225014 mutant genotypes (AG and GG) demonstrated a significantly lower EQ-5D index compared to those with the wild-type genotype (AA) (P = 0.025). Conversely, no statistically significant differences were observed in the EQ-5D Visual Analog Scale (VAS) scores or the individual domain scores of the EQ-5D-3L. The lack of statistical significance in the VAS and individual domain scores may be attributable to the limited sample size, which could have constrained the statistical power to detect differences. The EQ-5D index, a composite preference-based measure derived from five health-related quality of life dimensions and weighted according to societal preferences, appears to be more sensitive in detecting subtle variations across these dimensions. In the total thyroidectomy plus ^131^I treatment group, multivariate linear regression analysis revealed a positive association between the *DIO2* rs225014 mutant genotypes (AG, GG) and L-T4 dose/weight (β = 0.244, *P* = 0.028), suggesting that patients with these genotypes may require higher L-T4 doses to achieve effective TSH suppression. However, no significant differences in L-T4 dose or dose/weight were observed between rs225014 genotypes in unadjusted analyses, indicating that the association may be modest and influenced by individual variability in factors such as L-T4 absorption, metabolism, or adherence. The *DIO2* rs225014 polymorphism is located in the coding region of the *DIO2* gene, which encodes the type 2 deiodinase enzyme responsible for converting T4 to the biologically active T3. This mutation might alter enzyme activity, potentially leading to reduced T3 production at the tissue level. For these patients, T3 and T4 combination therapy may contribute to alleviate hypothyroidism symptoms, such as fatigue and malaise, improve quality of life, and enhance adherence to TSH suppression therapy [25, 26]. However, given that this hypothesis was not directly tested in our study, we propose it as a direction for future research rather than a definitive clinical recommendation. In postoperative PTC patients, the success of TSH suppression therapy was not only linked to tumor recurrence but also to quality of life and associated complications [18, 19]. Testing for the *DIO2* gene polymorphism may help determine whether L-T4 combined with low-dose T3 therapy would optimize treatment outcomes. By accumulating data on the postoperative management of PTC patients, this study aimed to provide more individualized treatment plans, improve quality of life, and reduce medication side effects, ultimately ensuring long-term benefits for these patients. Contrasted with the findings for *DIO2* rs225014, no significant associations were observed for *DIO2* rs12885300 (AA, AG vs. GG) in either group. Several factors may contribute to these negative results. The rs12885300 polymorphism (ORFa-Gly3Asp) is located in the 5’ untranslated region of the *DIO2* gene and may have a less direct impact on type 2 deiodinase enzyme activity compared to rs225014 (Thr92Ala), which is a missense mutation in the coding region that can alter enzyme function [14, 24]. Its hypothesized effect on the hypothalamic-pituitary-thyroid axis set point [15] may be less pronounced in post-surgical PTC, where L-T4 dominates homeostasis. Genetic or environmental factors may further obscure effects. The larger AA + AG subgroup suggests a potentially smaller effect size, requiring larger samples for detection.

This study had some limitations. The sample size is relatively small for genetic studies, and due to the small sample size, in mutation-carrying genotypes, homozygote and heterozygote were combined in the statistical analysis to ensure validity and reduce the impact of extreme values. Combining heterozygotes and homozygotes for analysis may mask genotype-specific effects However, this approach limited the ability to distinguish significant differences between heterozygous and homozygous individuals and may mask genotype-specific effects. It still need future research with larger cohorts to more robustly evaluate the clinical significance of *DIO2* polymorphisms and its role in thyroid hormone metabolism and patient outcomes. Several factors, such as patient compliance, drug interactions, comorbidities and dietary iodine intake, can influence L-T4 dose requirements and quality of life. We attempted to control for some of these factors during patient recruitment and data collection. However, due to the complexity of the clinical situation and the availability of certain clinical data, some factors were not included in the study. These confounding variables may potentially impact the trial results. L-T4 monotherapy was used during the whole TSH suppression therapy. While in some patients with the *DIO2* rs225014 mutation, poor TSH suppression or low self-reported quality of life was observed. However, we did not adjust the treatment regimen to include T4 and T3 combination therapy, nor did we assess whether this combination could improve TSH control and quality of life for these patients. We will further adjust and improve the treatment regimen in subsequent follow-ups and studies.

## Conclusion

In our research, among patients with papillary thyroid carcinoma (PTC), those in the lobectomy group exhibited higher quality of life compared to the total thyroidectomy plus 131I treatment group. In the lobectomy group, where a portion of the thyroid tissue was retained, the *DIO2* rs225014 and *DIO2* rs12885300 polymorphisms did not significantly affect quality of life, thyroid hormone levels, or L-T4 dose/weight. In the total thyroidectomy plus ^131^I treatment group, *DIO2* rs225014 mutant genotypes (AG, GG) showed a modestly lower EQ-5D index score and a slight association with higher L-T4 dose/weight ratios. No significant differences were found for *DIO2* rs12885300. These findings, limited by sample size and effect size, require validation in larger, multicenter studies.

## Supplementary Information

Below is the link to the electronic supplementary material.

Supplementary Material 1

## Data Availability

The data that support the findings of this study are not publicly available due to their containing information that could compromise the privacy of research participants. The data are available from the corresponding author upon reasonable request.

## Abbreviations

PTC: Papillary thyroid carcinoma
L-T4: Levothyroxine (T4, 3,5,3′,5′-triiodo-L-thyronine)
D1: Deiodinase type 1
D2: Deiodinase type 2
D3: Deiodinase type 3
DIO2: gene of type 2 deiodinase
TSH: Thyroid Stimulating Hormone
SNP: Single nucleotide polymorphisms
FT4: Free thyroxine
FT3: Free triiodothyronine
TPOAb: Thyroidperoxidase antibodies
TG: Thyroglobulin
EQ-5D-3L: EuroQol Five Dimensions 3 Level Questionnaire
EQ VAS: EuroQol Five Dimensions visual analogue scale
