# Supplementary material for: Impact of DIO2 polymorphisms on quality of life and TSH suppression therapy in patients with papillary thyroid cancer
Source: BMC Endocr Disord. 2025 Dec 2;25:278. doi: 10.1186/s12902-025-02085-x (PMC12673731; doi:10.1186/s12902-025-02085-x)
Supplement: Supplementary file 1 — Supplementary Material 1 [file 12902_2025_2085_MOESM1_ESM.docx]

| **EQ-5D-3L Paper Self-Complete** | |
| --- | --- |
| **Under each heading, please tick the ONE box that best describes your health TODAY.** | |
| **MOBILITY** |  |
| I have no problems in walking about | **□** |
| I have some problems in walking about | **□** |
| I am confined to bed | **□** |
| **SELF-CARE** |  |
| I have no problems with self-care | **□** |
| I have some problems washing or dressing myself | **□** |
| I am unable to wash or dress myself | **□** |
| **USUAL ACTIVITIES (e.g. work, study, housework, family or leisure activities)** |  |
| I have no problems with performing my usual activities | **□** |
| I have some problems with performing my usual activities | **□** |
| I am unable to perform my usual activities | **□** |
| **PAIN / DISCOMFORT** |  |
| I have no pain or discomfort | **□** |
| I have moderate pain or discomfort | **□** |
| I have extreme pain or discomfort | **□** |
| **ANXIETY / DEPRESSION** |  |
| I am not anxious or depressed | **□** |
| I am moderately anxious or depressed | **□** |
| I am extremely anxious or depressed | **□** |

**Table S1 EQ-5D-3L**

EQ-5D index is derived by applying a formula that essentially attaches values (weights) to each of the levels in each dimension. The index can be calculated by deducting the appropriate weights from 1, the value for full health (i.e. state 11111). The collection of index values (weights) for all possible EQ-5D states is called a value set. Most EQ-5D value sets have been obtained from a standardised valuation exercise, in which a representative sample of the general population in a country/region is asked to place a value on EQ-5D health states using methods such as the time trade-off (TTO) valuation technique or the VAS valuation technique. This approach ensures that the values represent the societal perspective. We utilize the value set constructed by Gordon G. Liu in 2022, which provides a set of social preference weights for China. The utility of 243 health states in the value set is shown in Table S2.

**Table S2 EQ-5D-3L value set**

| **State** | **Utility** |  | **State** | **Utility** |  | **State** | **Utility** |  | **State** | **Utility** |  | **State** | **Utility** |
| --- | --- | --- | --- | --- | --- | --- | --- | --- | --- | --- | --- | --- | --- |
| 11111 | 1 |  | 21222 | 0.562 |  | 11233 | 0.427 |  | 22232 | 0.321 |  | 13233 | 0.188 |
| 11112 | 0.859 |  | 12222 | 0.560 |  | 21313 | 0.427 |  | 23213 | 0.319 |  | 23313 | 0.188 |
| 11211 | 0.847 |  | 21213 | 0.558 |  | 12313 | 0.425 |  | 21323 | 0.317 |  | 33311 | 0.186 |
| 21111 | 0.832 |  | 12213 | 0.556 |  | 31311 | 0.425 |  | 33211 | 0.317 |  | 23232 | 0.185 |
| 12111 | 0.830 |  | 31211 | 0.556 |  | 21232 | 0.424 |  | 12323 | 0.315 |  | 13323 | 0.179 |
| 11121 | 0.823 |  | 11223 | 0.549 |  | 12232 | 0.422 |  | 31321 | 0.315 |  | 23322 | 0.176 |
| 11212 | 0.773 |  | 22122 | 0.545 |  | 22312 | 0.422 |  | 22322 | 0.312 |  | 23133 | 0.173 |
| 21112 | 0.758 |  | 22113 | 0.541 |  | 13213 | 0.420 |  | 13223 | 0.310 |  | 32223 | 0.171 |
| 12112 | 0.756 |  | 32111 | 0.539 |  | 11323 | 0.418 |  | 22133 | 0.309 |  | 33131 | 0.171 |
| 11122 | 0.749 |  | 21123 | 0.534 |  | 23212 | 0.417 |  | 32131 | 0.307 |  | 13332 | 0.155 |
| 21211 | 0.746 |  | 22221 | 0.533 |  | 21322 | 0.415 |  | 23222 | 0.307 |  | 31233 | 0.152 |
| 11113 | 0.745 |  | 12123 | 0.532 |  | 12322 | 0.413 |  | 11333 | 0.296 |  | 32313 | 0.150 |
| 12211 | 0.744 |  | 31121 | 0.532 |  | 21133 | 0.412 |  | 23123 | 0.295 |  | 32232 | 0.147 |
| 11221 | 0.737 |  | 11313 | 0.528 |  | 12133 | 0.410 |  | 21332 | 0.293 |  | 33213 | 0.145 |
| 22111 | 0.729 |  | 11232 | 0.525 |  | 31131 | 0.410 |  | 33121 | 0.293 |  | 31323 | 0.143 |
| 21121 | 0.722 |  | 21312 | 0.525 |  | 13222 | 0.408 |  | 12332 | 0.291 |  | 32322 | 0.138 |
| 12121 | 0.720 |  | 12312 | 0.523 |  | 22132 | 0.407 |  | 13313 | 0.289 |  | 32133 | 0.135 |
| 11311 | 0.700 |  | 13212 | 0.518 |  | 23113 | 0.405 |  | 13232 | 0.286 |  | 33222 | 0.133 |
| 11131 | 0.685 |  | 11322 | 0.516 |  | 33111 | 0.403 |  | 23312 | 0.286 |  | 23331 | 0.128 |
| 13111 | 0.678 |  | 11133 | 0.513 |  | 13123 | 0.396 |  | 32213 | 0.281 |  | 33123 | 0.121 |
| 21212 | 0.672 |  | 21132 | 0.510 |  | 22231 | 0.395 |  | 13322 | 0.277 |  | 31332 | 0.119 |
| 12212 | 0.670 |  | 12132 | 0.508 |  | 11332 | 0.394 |  | 13133 | 0.274 |  | 33312 | 0.112 |
| 11222 | 0.663 |  | 13113 | 0.506 |  | 23122 | 0.393 |  | 31223 | 0.274 |  | 33132 | 0.097 |
| 11213 | 0.659 |  | 23112 | 0.503 |  | 13312 | 0.387 |  | 23132 | 0.271 |  | 22333 | 0.092 |
| 22112 | 0.655 |  | 21231 | 0.498 |  | 22321 | 0.386 |  | 32222 | 0.269 |  | 32331 | 0.090 |
| 21122 | 0.648 |  | 12231 | 0.496 |  | 31213 | 0.384 |  | 22331 | 0.264 |  | 23233 | 0.087 |
| 12122 | 0.646 |  | 22311 | 0.496 |  | 23221 | 0.381 |  | 23231 | 0.259 |  | 33231 | 0.085 |
| 21113 | 0.644 |  | 13122 | 0.494 |  | 32212 | 0.379 |  | 32123 | 0.257 |  | 23323 | 0.078 |
| 22211 | 0.643 |  | 23211 | 0.491 |  | 13132 | 0.372 |  | 31313 | 0.253 |  | 33321 | 0.076 |
| 12113 | 0.642 |  | 21321 | 0.489 |  | 31222 | 0.372 |  | 31232 | 0.250 |  | 13333 | 0.057 |
| 31111 | 0.642 |  | 12321 | 0.487 |  | 32113 | 0.367 |  | 23321 | 0.250 |  | 23332 | 0.054 |
| 21221 | 0.636 |  | 31212 | 0.482 |  | 21331 | 0.367 |  | 32312 | 0.248 |  | 32233 | 0.049 |
| 11123 | 0.635 |  | 13221 | 0.482 |  | 12331 | 0.365 |  | 33212 | 0.243 |  | 32323 | 0.040 |
| 12221 | 0.634 |  | 22131 | 0.481 |  | 31123 | 0.360 |  | 31322 | 0.241 |  | 33223 | 0.035 |
| 11312 | 0.626 |  | 31113 | 0.470 |  | 13231 | 0.360 |  | 31133 | 0.238 |  | 31333 | 0.021 |
| 22121 | 0.619 |  | 11331 | 0.468 |  | 23311 | 0.360 |  | 32132 | 0.233 |  | 32332 | 0.016 |
| 11132 | 0.611 |  | 23121 | 0.467 |  | 32122 | 0.355 |  | 33113 | 0.231 |  | 33313 | 0.014 |
| 13112 | 0.604 |  | 32112 | 0.465 |  | 31312 | 0.351 |  | 13331 | 0.229 |  | 33232 | 0.011 |
| 11231 | 0.599 |  | 13311 | 0.461 |  | 13321 | 0.351 |  | 22233 | 0.223 |  | 33322 | 0.002 |
| 21311 | 0.599 |  | 22222 | 0.459 |  | 22223 | 0.345 |  | 32231 | 0.221 |  | 33133 | −0.001 |
| 12311 | 0.597 |  | 31122 | 0.458 |  | 23131 | 0.345 |  | 33122 | 0.219 |  | 23333 | −0.044 |
| 13211 | 0.592 |  | 22213 | 0.455 |  | 32221 | 0.343 |  | 22323 | 0.214 |  | 33331 | −0.046 |
| 11321 | 0.590 |  | 32211 | 0.453 |  | 31132 | 0.336 |  | 32321 | 0.212 |  | 32333 | −0.082 |
| 21131 | 0.584 |  | 21223 | 0.448 |  | 33112 | 0.329 |  | 23223 | 0.209 |  | 33233 | −0.087 |
| 12131 | 0.582 |  | 12223 | 0.446 |  | 21233 | 0.326 |  | 33221 | 0.207 |  | 33323 | −0.096 |
| 23111 | 0.577 |  | 13131 | 0.446 |  | 12233 | 0.324 |  | 21333 | 0.195 |  | 33332 | −0.120 |
| 22212 | 0.569 |  | 31221 | 0.446 |  | 22313 | 0.324 |  | 12333 | 0.193 |  | 33333 | −0.218 |
| 31112 | 0.568 |  | 22123 | 0.431 |  | 31231 | 0.324 |  | 31331 | 0.193 |  |  |  |
| 13121 | 0.568 |  | 32121 | 0.429 |  | 32311 | 0.322 |  | 22332 | 0.190 |  |  |  |
